# A Novel Biallelic Variant in *IHH* Causing Acrocapitofemoral Dysplasia in a Pakistani Family

**DOI:** 10.1002/mgg3.70085

**Published:** 2025-03-06

**Authors:** Tayyaba Saeed, Nousheen Bibi, Ashfaq Ahmad, Saadullah Khan, Muhammad Ansar, Naveed Wasif, Umm‐e‐ Kalsoom

**Affiliations:** ^1^ Department of Biochemistry Hazara University Mansehra Pakistan; ^2^ Department of Bioinformatics Shaheed Benazir Bhutto Women University Peshawar Pakistan; ^3^ Department of Bioinformatics Hazara University Mansehra Pakistan; ^4^ Department of Biotechnology and Genetic Engineering Kohat University of Science and Technology Kohat Pakistan; ^5^ Department of Biochemistry Quaid‐i‐Azam University Islamabad Pakistan; ^6^ Institute of Human Genetics, Ulm University and Ulm University Medical Center Ulm Germany; ^7^ Institute of Human Genetics, University Hospital Schleswig‐Holstein Kiel Germany

**Keywords:** acrocapitofemoral dysplasia, autosomal recessive, dynamic simulation, *IHH*, missense variant, Pakistan

## Abstract

**Background:**

Acrocapitofemoral dysplasia (ACFD) is a rare autosomal recessive disorder, characterized by postnatal onset of disproportionate short stature with short limbs, brachydactyly, cone‐shaped epiphysis, narrow thorax, and relatively large head. To date, only three homozygous missense mutations have been reported in the signaling amino terminal domain (201–308 amino acids) of the *IHH* gene in three ACFD families from Belgian, Dutch, and Turkish ethnicities.

**Methods:**

In the present study, we have investigated two patients in a Pakistani family affected with ACFD. Whole exome sequencing (WES) followed by Sanger sequencing was carried out for mutational screening. The variant was further validated by in silico modeling and molecular dynamics simulation analysis.

**Results:**

Data analysis revealed a novel homozygous missense variant [c.518C>A; p.(Ala173Asp)] in exon 2 of the *IHH* (NM_002181.4) gene. The variant segregated within the family and was not observed in unaffected ethnically matched controls. In silico modeling and dynamic simulation analysis revealed that the variant disturbed the core structure of the domain and destabilized the loop region and the region surrounding the variant.

**Conclusion:**

This study reports the first case of ACFD from Pakistan and identifies the fourth novel missense variant in the *IHH* gene that led to the broadening of the phenotypic and genotypic spectrum of ACFD.

## Graphical abstract

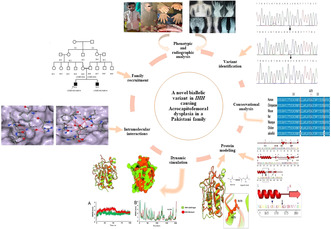

## 1 Introduction

Acrocapitofemoral dysplasia (ACFD; MIM 607778) is a rare autosomal recessive disorder characterized by postnatal onset disproportionate short stature of variable degrees with short limbs, brachydactyly, short broad nails, narrow thorax, pectus deformities, and a relatively large head, with an average IQ (Mortier et al. 2003; Hellemans et al. 2003). The distinguishing radiographic features are the cone‐shaped epiphyses of the hands, proximal femur, and to a variable degree at the shoulders, knees, and ankles in early childhood. The egg‐shaped femoral head with a short femoral neck and shortened tubular bones in the hands especially middle phalanges, are due to early prepubertal closure of the growth plate (Hellemans et al. 2003). In rare cases, radial bowing/angulation and genu varum are reported (Mortier et al. 2003; Cubuk and Duz 2021). A single flexion line of the bilateral fifth digit on the hands and feet has also been reported (Hellemans et al. 2003).

Homozygous mutations in Indian Hedgehog (*IHH*; MIM 600726) gene are responsible for causing ACFD (Hellemans et al. 2003) while Brachydactyly type A1 (BDA1; MIM 112500) presenting shortened or absent middle phalanges and often accompanied by short stature, is caused by heterozygous mutations in the same gene (Gao et al. 2001; Lodder et al. 2008). *IHH* is located on 2q35 and is composed of three exons that span 6 kbps and code for a member of the hedgehog (Hh) family of proteins that induces the differentiation of osteoblasts in the perichondrium during endochondral ossification (Yang et al. 2015). IHH is synthesized by early hypertrophic chondrocytes during the endochondral bone developmental process, leaving the proliferative pool. IHH peptide (45 kDa) is transported to the endoplasmic reticulum and golgi apparatus, where it undergoes autocatalytic internal cleavage into two domains, that is, a critically functional amino‐terminal (20 kDa) that possesses reported signaling activity and a 25 kDa domain essential for autoproteolytic processing at the C‐terminus (Liu et al. 2006).

The Hh signal after being synthesized as a membrane‐bound protein with cholesterol and palmitate modifications (Ma et al. 2002) is extracted by the Dispatched homologue 1 (DISP1) and transferred to the carrier protein that enables the extracellular mobilization of the Hh signal at long range by protecting the hydrophobic lipid modifications (Rohatgi and Scott 2007). In the absence of Hh ligand, PTCH1 restrains the activity of a 7‐pass TM protein, SMO (Smoothened) that causes the phosphorylation of GLI2 and GLI3 transcription factors, converting them to their corresponding repressor forms (GLIR). Hh binding to PTCH1 relieves SMO inhibition that in turn prevents the proteolytic processing of the GLI proteins, enabling them to become transcriptional activators (GLIA) and initiate the downstream signaling pathways (Zhang and Beachy 2023).

Up till now, only six cases of ACFD have been reported from Belgian, Dutch (Mortier et al. 2003; Hellemans et al. 2003), and Turkish families (Cubuk and Duz 2021). In the present study, we have presented the first case of ACFD from the Pakistani population. Whole exome sequencing (WES) of the family identified a novel homozygous missense variant [c.518C>A: p.(Ala173Asp)] in the *IHH* gene that segregated with the disease phenotypes in the family.

## 2 Materials and Methods

### 2.1 Sample Selection and DNA Extraction

**FIGURE 1:**
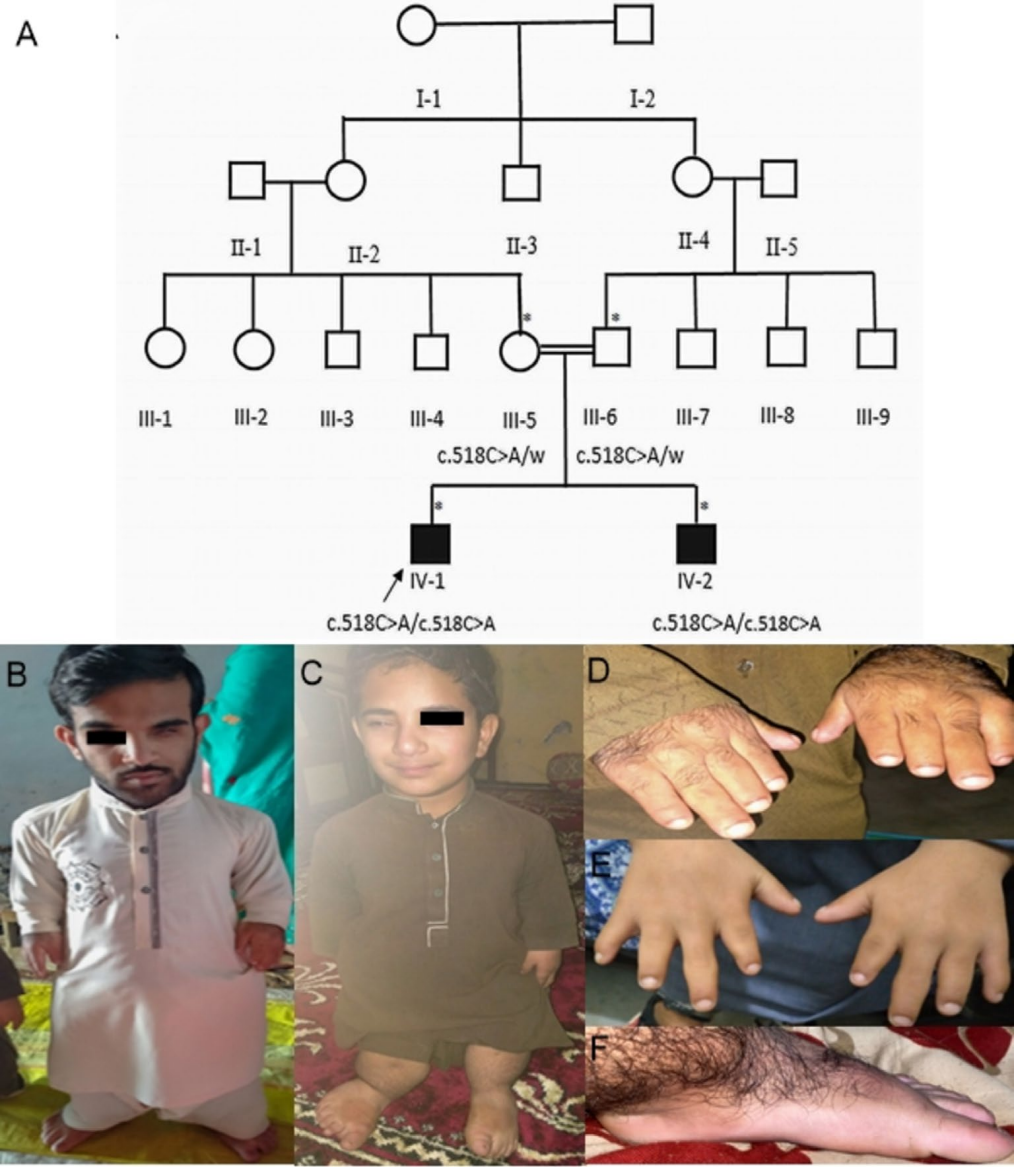
(A) Pedigree drawing indicating the autosomal recessive ACFD. Individuals available for the study are indicated by asterisk. (B) IV‐1 and (C) IV‐2 depicting severe short stature and unilateral ptosis. (D, E) Hands of IV‐1 and IV‐2 showing short hands and brachydactyly. (F) Foot of IV‐1 showing pes planus and hypertrichosis.

The Institutional Review Board and Bioethical Committee of Hazara University, Mansehra, Khyber Pakhtunkhwa, Pakistan approved (F.No. 73/HU/ORIC/2021/808) the study. Family elders were interviewed to construct the four‐generation pedigree (Figure 1A). In total, two unaffected members (III‐9, III‐10) and two affected members (IV‐1, IV‐2) participated in the study. Informed consent was obtained from the participating family members or their elders prior to the blood sampling and taking the photographs. Genomic DNA was extracted from the peripheral blood samples using a standard method (Sambrook et al. 1989).

### 2.2 Whole Exome Sequencing

WES was carried out on an affected individual (IV‐1) of the family that required exome capturing using the xGen Exome Research Panel v2 (Integrated DNA Technologies, Coralville, Iowa, USA). Sequencing by NovaSeq 6000 (Illumina, San Diego, CA, USA) was followed by alignment against the reference genome (GRCh37) and the Revised Cambridge Reference Sequence (rCRS) of the mitochondrial genome. EVIDENCE was used for Variant interpretation based on the recommended guideline (Richards et al. 2015) by considering the patient's phenotype, family history, and previous test results (Seo et al. 2020).

### 2.3 Segregation Analysis of the Variant

Sanger sequencing was carried out in affected individuals and their parents using the flanking primers (Table S1) designed from the intronic regions through primer 3 software (Rozen and Skaletsky 2000). A 50 μL reaction mixture was prepared and amplified using an initial denaturation of 95°C for 1 min, 35 cycles comprising denaturation at 95°C for 1 min, annealing at 57°C for 1 min, and elongation at 72°C for 1 min, followed by a final extension at 72°C for 10 min. The amplicons were purified using the Favorprep (FAGCK 001–1) kit prior to sequencing.

### 2.4 *In Silico* Modelling of IHH

Experimentally determined three dimensional structure IHH N‐terminal signaling domain (41aa‐195aa; PDBID:3K7G) was obtained from RCSB protein data bank (https://www.rcsb.org/). To access the structural impact of identified variant (p.Ala173Asp) in IHH protein, a mutant model was build using AlphaFold version 2, through Colab setup. Total of five structures were generated, with custom template options (Mirdita et al. 2022). The mmseq2 package, with uniref_env was considered and alignment was generated in unpaired mode (Steinegger and Soding 2017). To predict protein in monomeric status, AlphaFold_ptm model was used for three recycles and random seed. Aside visual folding inspections of the generated models, pLDDT scores were evaluated and compared. A mutant IHH structure (IHH^A173D^) with highest accuracy interpreted by pLDDT > 97.4% was achieved. UCSF Chimera 1.5.6 (Meng et al. 2006) was used for energy minimization and structure refinements.

### 2.5 Molecular Dynamic Simulations

Molecular dynamics simulations of wild‐type (IHH^WT^) and mutant (IHH^A173D^) were performed using the GROMACS package (Spoel et al. 2005). A periodic cubic box was created for both the mutant and wild‐type systems, including solvent molecules and neutralized with Na + Cl counter ions. The systems were simulated under 1 bar constant pressure and 300 K temperature conditions for 100 ns. VMD (Humphrey et al. 1996), PyMol (http://www.pymol.org), and GROMACS tools were used to analyze the stability and fluctuations of the simulated systems.

## 3 Results

### 3.1 Clinical Evaluation

Both the affected individuals were born as a result of a consanguineous union without any complications in full‐term pregnancy. At the time of sampling, IV‐1 was 20 years old, having a height of 102 cm (−10.4 SD) height and IV‐2 was 14 years old, having a height of 84 cm (−9.6 SD) Both affected individuals had severe short stature, short limbs, a narrow thorax, and a relatively large head (Figure 1B,C). Their hands were short, with brachydactyly and short nails (Figure 1D,E). Some novel phenotypes, like unilateral ptosis (Figure 1B,C) pes planus, and hypertrichosis (Figure 1F) were also observed in our patients. The height of the father (53 years old) and the mother (45 years old) was short, with 155 cm (−3.0SD) and 140.2 cm (−3.6 SD), respectively, but their hands were normal, with normal fingers.

**FIGURE 2:**
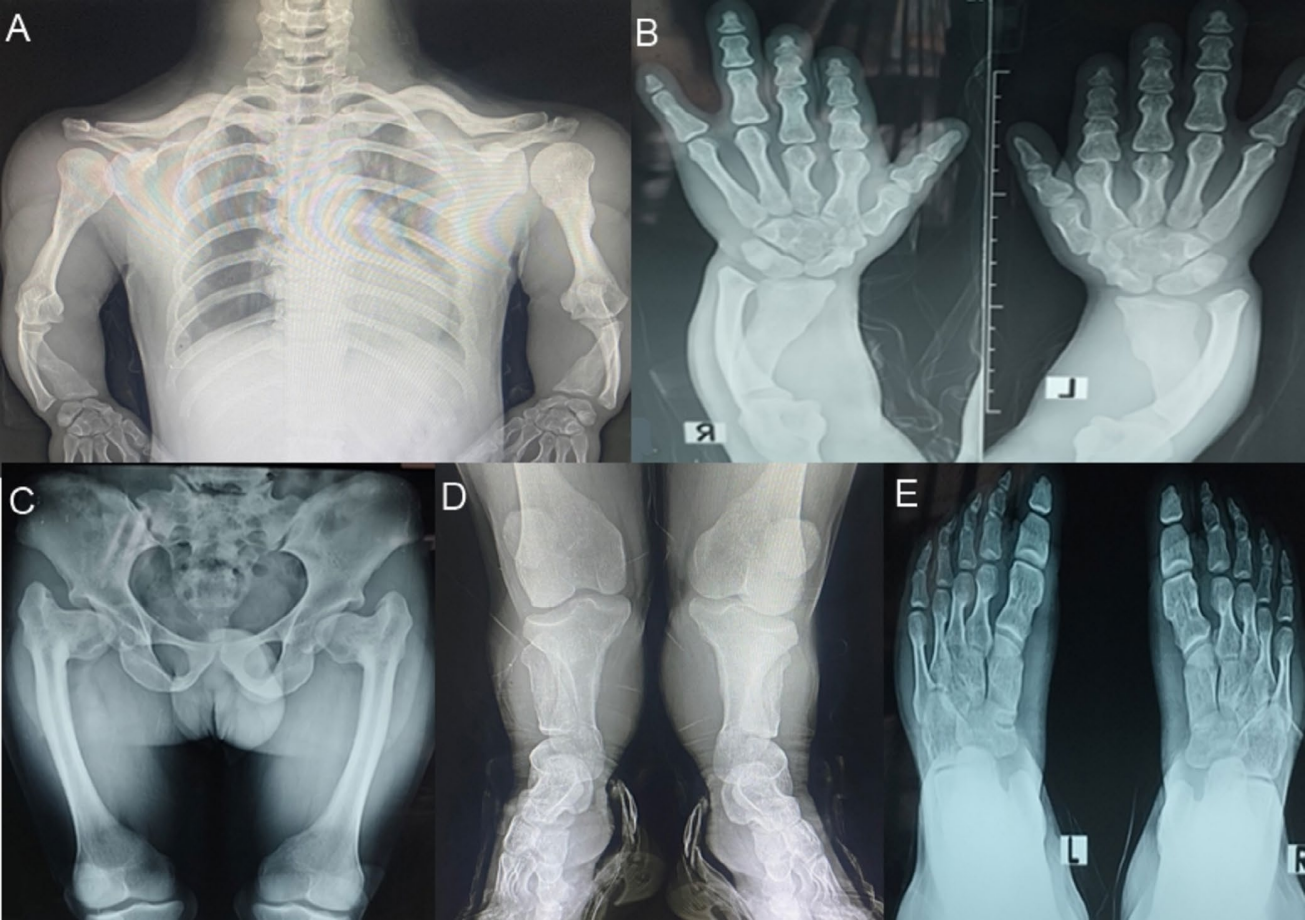
(A) Chest radiographs of IV‐1 showing narrow thorax, short ribs and clavicles. Short humerus bone with cone shaped epiphysis at the proximal end. Elbow joints depicting dislocated radial head and increased inclination angle of humerus head. Bilateral Varus angulation at the radial and ulnar bones with medlung like deformity at the distal radioulnar joint. (B) Hands radiographs indicating hypoplasia of middle phalange of little finger, fusion of middle and distal phalanges of 1st and 2nd finger and thumb, shorter metacarpal bones, and subluxation of ulnocarpal joint. (C) Pelvic region showing dysplastic acetabulum coxa vara with greater traochanter over growth, lateral pelvis tilted to right side, egg‐shaped capito femoral epiphyses with a short femoral neck, thin femoral shaft with narrow medullary cavity, hyperplastic distal femur, and genu vara (D) Extremely short tibia and fibulae with irregular proximal tibial metaphysis and cone shaped distal tibial epiphysis (E) short metatarsals with conical tear drop epiphysis, accessory navicle foot deformity, and fused middle and distal phalanx of fifth toe bilaterally.

Chest radiographs of IV‐1 revealed a narrow thorax, short ribs and clavicles, lumbar lordosis, and cupping at the costal ends of the ribs. The humerus bone was severely shortened with cone‐shaped proximal epiphysis. A dislocated radial head with an increased inclination angle of the humeral head was seen at the elbow joints (Figure 2A). All the carpal bones were fused, and the radial and ulnar bones were short with bilateral varus angulation and medlung‐like deformity at the distal radioulnar joint. Also, the subluxation of the ulnocarpal joint was clearly evident in the hand radiograph. The 1st, 2nd, and 3rd metacarpals were short compared to the 4th and 5th metacarpals, leading to the shortening of the thumb, index, and middle fingers. The proximal phalanges were smaller in size in the index finger; the middle phalanges had a short and stubby appearance, and the middle and distal phalanges of the 1st finger, 2nd finger, and thumb were fused (Figure 2B).

Pelvic examination showed bilateral osteoarthritis, short and flared iliac wings, dysplastic acetabulum, coxa vara with greater trochanter overgrowth. The capital femoral epiphyses had egg‐shaped appearances with a short femoral neck. The femur bone was short with a short neck, thin shaft, and narrow medullary cavity and hyperplastic distal end. The knee joint had genu vara (Figure 2C) and the tibia and fibula bones were extremely short with irregular proximal tibial metaphyses and cone‐shaped distal tibial epiphyses. Foot examination revealed short and broad metatarsals with conical tear drop epiphyses, accessory navicular foot deformity, and fused middle and distal phalanx of the fifth toe bilaterally (Figure 2D).

### 3.2 Variant Identification

**FIGURE 3:**
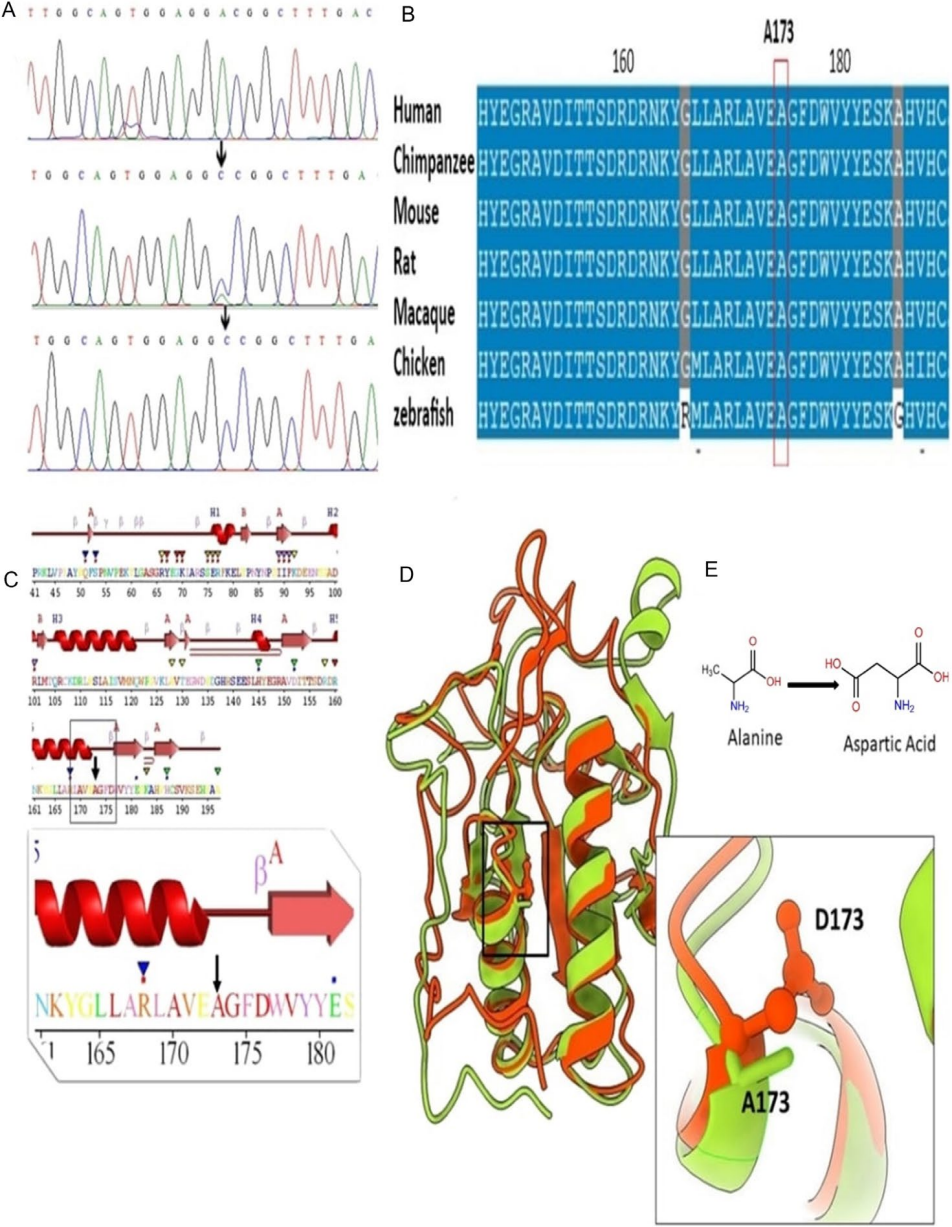
(A) Sequencing chromatograms of the variant (c.518C>A) identified in an affected (upper panel), a carrier (middle panel) and an unaffected (lower panel). (B) Conservation analysis of wild‐type residue. (C) Secondary structure elements indicating the loops, sheets and helices along with conservation profile of the residues. Mutant residue is highly conserved. (D) Superimposition of IHH^WT^ (green) and IHH^A173D^ (orange) proteins in ribbon representation with residues shown in stick model. (E) 2D representation of wild‐type (Ala) and mutant (Asp) residue.

In total, 67,851 single nucleotide variants (SNV) and 11,337 small indels were identified, of which the novel homozygous missense variant (c.518C>A) in the *IHH* (NM_002181.4) gene was considered clinically significant and was interpreted as deleterious or damaging by various In silico tools (Table 1). The parents were heterozygous carriers, while both affected individuals carried the homozygous mutant allele (Figure 3A). This variant caused the substitution of Alanine with Aspartate at amino acid position 173 (NP_002172.2: p.(Ala173Asp)) in the IHH protein. Conservation analysis showed that the wild‐type residue is highly conserved and that the variant at this site is probably damaging to the protein (Figure 3B). The sequence variant was classified as likely pathogenic (Table S2) based on ACMG criteria (Richards et al. 2015).

**TABLE 1:** *I* *n silico* analysis of the identified variant using various Bioinformatics tools.

Bioinformatic tools	Score	Prediction
SIFT	0	Deleterious
PhD‐SNP	7	Disease causing
SNPs&GO	Nil	Disease causing
MutPred	0.878	Altered
PREDICT SNP	87	Deleterious
PolyPhen	0.997	Probably damaging
REVEL	0.96	Damaging effect
3Cnet	0.99	Damaging effect

### 3.3 2D and 3D Structure of IHH Protein

The secondary structure elements of the amino signaling domain are arranged into six loops and seven sheets (β1–β7) of variable length that are accompanied by two proper helices (α1 and α2) facing each other in a parallel conformation. The corresponding variant p.(Ala173Asp) is located at the extreme C‐terminal position of α2 helix (Figure 3C). Superimposition of IHH^WT^ and IHH^A173D^ showed differences in the 3D structure of the mutant model with an RMSD value of 0.93 Å (Figure 3D,E). The wild‐type Ala residue is situated deep within the domain's core, and its substitution with the mutant Asp disrupted the structural integrity of the core owing to the difference in size and charge of these residues. Resultantly, the hydrophobic interactions were lost within the core, which interfered with the proper folding of theprotein.

### 3.4 Molecular Dynamic Simulation of Wild‐Type and Mutant IHH Protein

**FIGURE 4:**
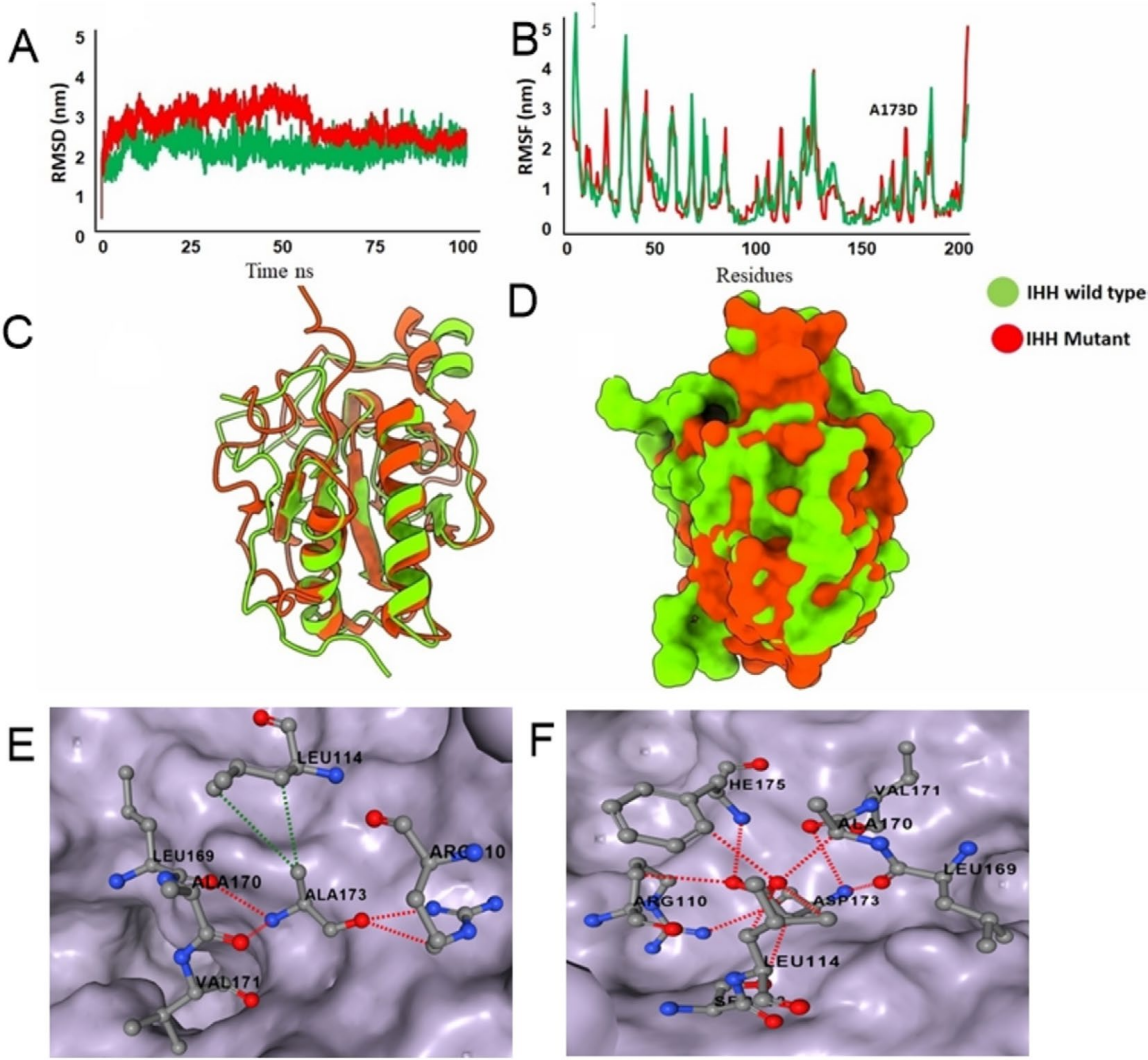
Molecular dynamic simulation of IHH^WT^ and IHH^A173D^. (A) RMSD (B) RMSF graph of wild‐type and mutant IHH system. (C, D) Represents the ribbon and surface representation of IHH^WT^ and IHH^A173D^ at 100 ns. (E, F) Intramolecular interactions of Ala173 (E) and Asp173 (F). Hydrophobic interactions are shown in green dotted line while hydrogen bonding is shown in red dotted lines.

In order to elucidate any impacts of p.(Ala173Asp) variant, a 100 ns simulation was performed both for IHH^WT^ and IHH^A173D^ proteins. In IHH,^wt^ RMSD analysis showed stability in the system throughout the simulations, with the RMSD value stable between ∼0.2 and ∼0.3 nm. In IHH,^A173D^ the RMSD starting from ∼0 nm showed fluctuation between ∼0.2 and ∼0.35 nm until 55 ns, with a decrease in stability, and the system got stabilized between ∼0.2 and ∼0.3 nm for the rest of the simulation experiment (Figure 4A). Root mean square fluctuation (RMSF) analysis of IHH^wt^ and IHH^A173D^ revealed overall stability, except in the loop region and the region surrounding the variant (Figure 4B). Superimposition of IHH^wt^ and IHH^A173D^ at 100 ns (Figure 4C,D) demonstrated that most of the fluctuations were present in the loop region. The wild type residue (Ala173) made several intramolecular interactions for stable protein conformation while mutant residue (Asp173) lost some interactions and made few new interactions (Figure 4E,F). This change resulted in decreased protein stability with ΔΔG energy of ‐1.026kcal/mol.

## 4 Discussion

*IHH* is one of the three mammalian homologues of the *D. melanogaster* segment polarity gene, Hh (Jeong and McMahon 2002) that is essential for angiogenesis, hematopoiesis as well as in skeleton formation (Mortier et al. 2003; Hellemans et al. 2003). Ihh signaling plays a crucial role in endochondral ossification, where its strong expression in prehypertrophic chondrocytes controls the bone growth through coordinated proliferation and differentiation of chondrocytes (Vortkamp et al. 1996; Long et al. 2004). Ihh stimulates the production of parathyroid hormone–related peptide (PTHrP) in the periarticular growth plate that slows down the switching from proliferation to differentiation. Thus, Ihh is a key player in determining the rate and site in the growth plate at which hypertrophic differentiation occurs (Karp et al. 2000). Mutations in the *IHH* gene cause various skeletal chondrodysplasias including ACFD, brachydactyly type A (Merchant and Matsui 2010; Vasques et al. 2018), and short stature (Vasques et al. 2018; Long et al. 2001). *IHH* locus duplications are associated with syndactyly and craniosynostosis (Klopocki et al. 2011). Up‐regulation of the Hh pathway is significantly associated with multiple forms of cancers along with its involvement in cancer stem cells maintenance (Ma et al. 2002).

To date, only three homozygous missense mutations [c.137C>T; p.(Pro46Leu), c.569T>C; p.(Val190Ala), c.478C>T; p.(Arg160Cys)] have been reported in the signaling amino terminal domain of the *IHH* gene in three ACFD families from Belgian, Dutch, and Turkish ethnicities (Hellemans et al. 2003; Cubuk and Duz 2021). Here, we report the first case of ACFD from Pakistan and identify the fourth novel missense variant (c.518C>A) in exon‐2 of the *IHH* (NM_002181.4) gene that substituted the highly conserved non‐polar Ala residue with a negatively charged Asp residue at the 173 amino acid position in the same functional IHH N‐terminal domain that is essential for the local and long‐range signaling (Porter et al. 1995; Fresquet et al. 2007). This variant is likely to disrupt the essential core structure of the protein domain due to the size and charge difference of the wild‐type and mutant residues, leading to a loss of hydrophobic interactions within the core, potentially causing protein folding problems. This was evident in the comparative RMSF analysis of the wild‐type and mutant proteins that revealed the instability in the loop region affecting the IHH binding to its receptor. The altered surface charge and flexibility of the helix loop may have disrupted the Hh signaling at multiple levels ranging from signal biogenesis to signal transduction and led to diminished IHH signaling in the growth plate. Ultimately, the rate of chondrocyte differentiation would have increased, causing early growth plate closure and bone shortening, as proposed by Hellemans et al. (2003).

The phenotypes of both the affected individuals in this study, including severe short stature, short limbs, large head, narrow thorax, small broad nails, lumbar lordosis, brachydactyly, and an average IQ, were the same as reported in all six previously reported ACFD patients (Mortier et al. 2003; Cubuk and Duz 2021). Despite the age difference among the ACFD patients reported to date (Table 2), early growth plate closure was evident in all of them, which led to the shortening of long tubular bones in the arms and legs, along with cone‐shaped epiphyses. Commonly reported radiographic features, that is, short and broad metacarpal and metatarsal bones with conical teardrop epiphyses, short middle phalanges, fused phalanges, coxa vara, short and flared iliac wings with dysplastic acetabulum, egg‐shaped capito‐femoral epiphyses with a short femoral neck, and hyperplastic distal femur, were also observed in our patients. Cubuk and Duz (2021) reported the fused middle and proximal phalanges of 5th finger and toes and an osteoporotic appearance in the spinal column; however, in the present study, the middle and distal phalanges of 1st finger, 2nd finger and thumbs were fused, and a bilateral osteoarthritic appearance was observed in the hip joint of IV‐1, which caused reduced joint mobility and function.

**TABLE 2:** Phenotypic comparison of patients reported in the present study with those reported from other ethnicities.

Phenotype/Genotype	Present study		Hellemans et al. (2003); Mortier et al. (2003)				Cubuk and Duz (2021)	
Origin of family	Pakistani		Belgian		Dutch		Turkish	
Variant	c.518C>A; p.(Ala173Asp)		c.137C>T; p.(Pro46Leu)		c.569C>T; p.(Val190Ala)		c.478C>T; p.(Arg160Cys)	
Patients	IV‐1	IV‐2	1	2	3	4	II‐3	II‐5
Age (years)	20	14	9.5	9.5	7	9	32	28
Height (cm)	102 (−10.4SD)	84 (−9.6 SD)	107 (−3.5SD)	121.5 (‐3SD)	97.5 (−7 SD)	97.5 (−7 SD)	120 (−7.3 SD)	110 (−8.9 SD)
Weight (kg)	30	20	25.4	26.5			−	−
Head circumference (centile)	71st	20th	3th–50th	50th–75th	90th	98th	50th	75th
Short limbs	+	+	+	+	+	+	+	+
Brachydactyly	+	+	+	+	+	+	+	+
Small broad nails	+	+	+	+	NR	NR	+	+
Short humerus	+	NA	+	+	+	+	+	+
Dislocated radial head	+	NA	NR	NR	NR	NR	NR	NR
Radial ulnal angulation	+	NA	−	−	−	−	+	+
Subluxation of ulnocarpal joint	+	NA	NR	NR	NR	NR	NR	NR
Cone shaped epiphysis	+	NA	+	+	+	+	−	−
Retarded/dislocated Carpal bone age	+	NA	+	+	+	+	−	−
Short metacarpal bones	+	+	+	+	+	+	+	+
Tear drop metacarpals	+	NA	−	−	+	+	NR	NR
Short middle phalange	+	+	+	+	+	+	+	+
Fusion of phalanges	Middle and distal in 1st and 2nd finger and thumb	NA	NR	NR	NR	NR	Middle and proximal in 5th fingers and toes	Middle and proximal in 5th fingers and toes
Short and flared Iliac wings	+	NA	−	−	+	+	−	NR
Thorax	Narrow	NA	Normal	Normal	Pectus craniatum	Pectus excavatum	Narrow	Narrow
Lumber lordosis	+	NA	+	+	−	+	−	+
Coxa vara	+	NA	−	+	+	+	−	−
Lateral pelvis tilted to right side	+	NA	NR	NR	NR	NR	NR	NR
Dysplastic acetabulum in pelvic region	+	NA	NR	NR	NR	NR	NR	NR
Egg shaped femoral head	+	NA	+	+	+	+	NR	NR
Short femoral neck	+	NA	+	+	+	+	+	+
Thin femoral shaft with narrow medullary cavity	+	NA	NR	NR	NR	NR	NR	NR
Hyperplastic distal femur	+	NA	+	−	−	+	−	−
Genu vara	+	+	+	−	+	−	−	−
Short tibia and fibula	+	NA	+	+	+	+	+	+
Short metatarsals	+	+	NR	NR	NR	NR	NR	NR
Pes planus	+	+	NR	NR	NR	NR	NR	NR
Hypertrichosis	+	−	NR	NR	NR	NR	NR	NR
Ptosis	+	+	NR	NR	NR	NR	NR	NR

*Note:* + indicates presence and – indicates absence.

Abbreviations: NA, not available; NR, not reported; SD, standard deviation.

Our proband (IV‐1) also had a rarely reported genu vara phenotype observed in only 1 of the 4 patients reported by Mortier et al. (2003) due to which he had a waddling walk. His younger brother (IV‐2) also had difficulty in walking and was dependent on parents for outdoor visits. Furthermore, some additional features like pes planus, hypertrichosis, and unilateral ptosis were also observed in our patients (Table 2). In conclusion, the current study reports the first case of ACFD from Pakistan and identifies the fourth novel homozygous missense variant (c.518C>A) in the *IHH* gene, hence broadening the phenotypic and genotypic spectrum of the disorder.

## Author Contributions

Tayyaba Saeed: conducted research, analyzed data, and wrote the main manuscript text; Nosheen Bibi and Ashfaq Ahmad: performed the bioinformatics analysis; Saadullah Khan: study design and approved the version to be published; Muhammad Ansar: co‐supervised the work and approved the manuscript; Naveed Wasif: data analysis and manuscript editing; Umm‐e‐Kalsoom: study design, research supervision, and manuscript evaluation. All authors reviewed the manuscript.

## Ethics Statement

The research study was approved by the Institutional Review Board (IRB) of Hazara University, Mansehra, Pakistan, under ethical committee approval number “F.No. 73/HU/ORIC/2021/808.”

## Consent

Informed written consent was taken from participating members of families. Blood sampling from unaffected and affected individuals was carried out according to guidelines provided by the Declaration of Helsinki.

## Conflicts of Interest

The authors declare no conflicts of interest.

## Supporting information


**Table S1.** Primers sequences used for amplification of the IHH gene.

**Table S2.** ACMG criteria for the classification of identified variant in IHH gene.

## Data Availability

We have submitted the novel variant to ClinVar (https://submit.ncbi.nlm.nih.gov/clinvar/) under an accession number SCV005061356.
