# Supplementary material for: A Novel Biallelic Variant in IHH Causing Acrocapitofemoral Dysplasia in a Pakistani Family
Source: Mol Genet Genomic Med. 2025 Mar 6;13(3):e70085. doi: 10.1002/mgg3.70085 (PMC11883292; doi:10.1002/mgg3.70085)
Supplement: Supplementary file 1 — Table S1. Primers sequences used for amplification of the IHH gene. [file MGG3-13-e70085-s002.docx]

###### Table S1: Primers sequences used for amplification of the *IHH* gene

| Exon no | Forward (5’→3’) | Reverse (5’→3’) | Product size (bp) | Temp(°C) |
| --- | --- | --- | --- | --- |
| 2 | CCTCCAGATTTGTGATACCG | CCTAGGCTCCTACCATGACA | 700bp | 57 |
