# Supplementary material for: A Novel Biallelic Variant in IHH Causing Acrocapitofemoral Dysplasia in a Pakistani Family
Source: Mol Genet Genomic Med. 2025 Mar 6;13(3):e70085. doi: 10.1002/mgg3.70085 (PMC11883292; doi:10.1002/mgg3.70085)
Supplement: Supplementary file 2 — Table S2. ACMG criteria for the classification of identified variant in IHH gene. [file MGG3-13-e70085-s001.docx]

| Gene | *IHH* |
| --- | --- |
| Variant | c.518C>A; p. (Ala173Asp) |
| **ACMG criteria pathogenic variant** | |
| **Very strong** | |
| PVS1-null variant |  |
| **Strong** |  |
| PS1-same AA as established pathogenic variant |  |
| PS2-denovo |  |
| PS3-*invitro* assay |  |
| PS4- increase prevalence of the variant |  |
| **Moderate** | |
| PM1- Mutational hotspot and/or critical and well-established functional domain | 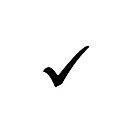 |
| PM3-cis/trans with pathogenic variant |  |
| PM4-Protein length change |  |
| PM5-same aa position, different change |  |
| PM6-assumed denovo |  |
| **Supporting** | |
| PM2-absent from controls | 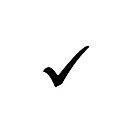 |
| PP1-cosegregation | 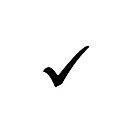 |
| PP2-low rate of benign missense variation | 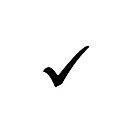 |
| PP3-computaional evidence support | 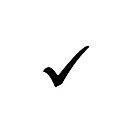 |
| PP4- patient phenotypes highly specific | 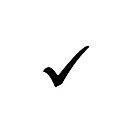 |
| PP5- reputable resource reports as pathogenic |  |

**Table S2**: ACMG criteria for the classification of identified variant in *IHH* gene
